# Prevalence of seropositivity of selected herpesviruses in patients with multiple sclerosis in the North of Jordan

**DOI:** 10.1186/s12883-020-01977-w

**Published:** 2020-10-29

**Authors:** Raid M. Kofahi, Hassan M. Kofahi, Suhib Sabaheen, Majdi Al Qawasmeh, Aiman Momani, Ahmed Yassin, Kefah Alhayk, Khalid El-Salem

**Affiliations:** 1 Department of Neurosciences, Jordan University of Science and Technology, P.O. Box 3030, Irbid, 22110 Jordan; 2 Department of Medical Laboratory Sciences, Jordan University of Science and Technology, Irbid, Jordan

**Keywords:** Multiple sclerosis, Autoimmunity, Varicella, Jordan, Middle East

## Abstract

**Background:**

Multiple sclerosis (MS) is a neurological disease that is caused by an autoimmune response that results in the neuron’s demyelination in the central nervous system. The exact etiology of MS is not clear; however, several environmental and genetic factors are believed to participate in its initiation and development, including exposure to viruses. This study aims to investigate the association between the seropositivity and antibody titer of selected herpesviruses and MS in Jordanian MS patients.

**Method:**

In this study, 55 MS patients and 40 age- and gender-matching apparently healthy volunteers were recruited from two main hospitals in the north of Jordan. MS patients were grouped into three types of MS based on the clinical presentation of the disease. Blood samples were collected from the participants and the IgG antibodies for human herpesvirus 6 (HHV-6), Epstein-Barr virus (EBV) nuclear antigen (EBNA), EBV viral capsid antigen (VCA) and varicella-zoster virus (VZV) were assayed by ELISA. The prevalence of seropositivity and the antibody level for each of the antibodies were compared between MS patients and controls and between the three types of MS.

**Results:**

There was no significant difference in the prevalence of seropositivity and in the levels of antibodies for HHV-6, EBNA and VCA between MS patients and controls and between the three types of MS. In contrast, the number of seropositive patients and the level of IgG antibodies for VZV were significantly higher in MS patients compared to the control.

**Conclusion:**

This study showed that patients with MS in the north of Jordan were more likely to be seropositive for VZV than the general population. Based on this finding, we recommend further studies to evaluate the seropositivity to VZV to be carried out in other parts of Jordan and the greater middle east to find out if there is a correlation between MS and previous infection with VZV.

**Supplementary Information:**

The online version contains supplementary material available at 10.1186/s12883-020-01977-w.

## Background

Multiple sclerosis (MS) is one of the most prevalent neurological diseases affecting approximately 2.1 million patients worldwide [1]. It is, widely, believed that MS is caused by an autoimmune response that affects the myelin sheath in the central nervous system, which subsequently results in the demyelination of nerve cells in the brain and the spinal cord [2]. The course of disease varies largely among MS patients, and can be presented as one of three clinical types [3, 4]. Most of the patients experience a relapsing-remitting MS (RRMS) pattern, which is characterized by periods of disease activity and symptoms that are followed by periods of partial or complete recovery [3, 5]. The disease may transform into secondary progressive MS (SPMS) over a period of about 20 years in about 70% of RRMS patient. The transformation into SPMS can occur at a rate of 2–3% per year [6]. SPMS is, currently, considered as the second phase of the disease. During this phase of the disease, patients continue to experience worsening of disease symptoms, without periods of remission [7]. Finally, approximately 10–15% of MS patients experience the primary progressive form of MS (PPMS), which is characterized by an unremitting reduction in neurological functioning that is not preceded by episodic relapses [8].

Similar to most of other autoimmune diseases, the exact etiology of MS is not fully understood. However, it is strongly believed that multiple genetic and environmental factors play a role in the initiation and progression of the disease. Exposure to viruses is thought to be a potential environmental factor. Viral infections had been linked to increased risk of the development of different autoimmune diseases. For example, it has been reported that enterovirus and cytomegalovirus (CMV) increase the risk of type 1 diabetes [9–13]. Infections with Epstein-Bar virus (EBV) and CMV increase the risk of systemic lupus erythematosus (SLE) [14–16]. Consequently, the association between viral infections and the development and progression of MS was investigated by several research groups in different geographical areas. These groups reported associations between MS and infections with several viruses, including EBV, CMV, and human herpesvirus 6 (HHV6) [17–19].

*Herpesviridae* is a family of enveloped, double-stranded DNA viruses, which is divided into three subfamilies; alpha, beta and gamma [20]. However, only a few of these viruses can infect humans. After an initial infection with a herpesvirus, the virus enters a latency phase in which the it remains dormant for an extended period of time. Reactivation of the virus later in life causes a recurrence in disease (reviewed in [21]). Human Herpesvirus 6 (HHV-6) is a member of the β-herpesviruses subfamily. HHV-6 was first isolated in 1986 from patients with AIDS and immunoproliferative syndrome, and was initially referred to as human B-lymphotropic virus (HBLV) [22]. However, subsequent studies revealed that this virus was actually able to infect a wide variety of organs including salivary glands, epithelial cells, T cells, and macrophages [23]. Epstein-Barr virus (EBV) is a member of the γ-herpesviruses subfamily that is able to infected B cells and epithelial cells [24]. EBV was first isolated in 1964 from a Burkitt’s lymphoma cell line [25]. EBV is now known to cause infectious mononucleosis and other diseases. Varicella-zoster virus (VZV) is classified as a member of alpha Herpesviruse subfamily. Primary infection with VZV causes varicella (chickenpox), after which the virus undergoes a state of latency in the dorsal ganglia [26]. Reactivation of the virus later in life causes a more severe disease called shingles [26].

Several previous studies reported that exposure to Herpesviruses may act as a trigger for the development of MS [27–32]. Despite these reports, the role of different viral infections and viral reactivation in MS development is still highly controversial. The reason for this controversy could be attributed in part to discrepancies between the results of published studies, which are probably confounded by variations in genetic and environmental factors between the different geographical locations in which the studies were conducted. It was this discrepancy that prompted us to investigate whether the infection and reactivation of Herpesviruses could be associated with the development and progression of MS in the Jordanian population. Such information could be particularly important in light of the fact that the association was not studied previously in Jordan. Identifying this association would be of great importance to understanding the risk factors for the development and progression of MS in Jordan, where etiological studies are scarce. It can also help predict the disease’s possible outcomes and select the best treatment options for the Jordanian MS patients.

## Methods

### Study sample

The study population was defined as all visitors of the outpatient clinics at King Abdullah University Hospital and Princess Basma Teaching Hospital in the period between July/2017 and November/2017. Ninety-five participants (55 MS patients, and 40 apparently healthy controls) were enrolled in the study. Ages for study participants ranged between 19 and 63 years. The study was approved by the institutional review board (IRB) at King Abdullah University Hospital and was also approved by the Jordanian Ministry of Health. All the participants signed an informed written consent before participating in the study. Participants in the study were matched on two factors; gender and age. The percent of male and female participants were closely matched between the control and the patient groups. In addition, the age range of 19–63 years that was defined in the inclusion criteria for this study was stratified into groups of 5-year periods, where a similar percentage of participants had to enroll in both groups within the different strata.

Patients’ information (age, gender and type of MS) were collected by using a questionnaire form that was developed by us for this study (see supplementary file 1) and by consulting their medical records. Patients were divided into three groups based on the type of MS. These groups are: RRMS, SPMS and PPMS. There were no exclusion criteria in this study in terms of age, sex, race or geographical residence. Gender- and age-matching healthy control group was recruited and used for comparison purposes.

### Blood sample collection and processing

For each participant, an approximately 4 ml blood sample was collected in a plain tube and was transported immediately to the research laboratory in the faculty of Applied Medical Sciences – Department of Medical Laboratory Sciences at Jordan University of Science and Technology (JUST). Blood samples were centrifuged upon arrival then serum was separated. Each serum sample was divided into aliquots into at least four small tubes and immediately stored at − 20 °C until the day of analysis.

### Measurement of antibody titers

HHV-6 IgG antibody was assayed by using a commercially available semi-quantitative ELISA kit (Vidia, Czech Republic). The protocol recommended by the manufacturer was followed. Index values for HHV-6 IgG were calculated by using the following formula: Index = the mean optical density (OD) value for the sample/cut-off value. In the qualitative analysis, index values above 1.1 were considered positive.

The IgG antibodies for Epstein Barr nuclear antigen (EBNA), EBV viral capsid antigen (VCA) and VZV were measured by commercially available quantitative enzyme-linked immunosorbent assay (ELISA) kits (IBL, Germany) according to the manufacturer’s recommended protocol. Any sample with antibody level above 12 U/ml was considered positive in the qualitative analysis.

### Statistical analysis

Data analysis was performed by Graph Prizm 7 software. Descriptive statistics were used to summarize patients’ demographics. Chi-square and two-tailed t-test were used to test for statistical differences between the patients’ group and the control group, and then between the three subgroups of MS. Analysis of variance (ANOVA) was used to test the antibody level differences between the three groups of MS patients. *P*-values of less than 0.05 were considered significant.

## Results

### Study population

A total of 55 MS patients and 40 controls were enrolled in the study. The two groups were matched in the percent of male-to-female ratio (*P* = 0.763). The control group consisted of 15 (37.5%) males and 25 (62.5%) females, while in the patients’ group, there were 20 (36.4%) males and 36 (62.5%) females (see Table 1). The difference in the enrolment among the two genders can be explained by the higher prevalence of MS among females than males. The control group’s mean age was 35.5 ± 10.8 years, and 36.2 ± 11.2 years for the MS group. There was no statistically significant difference in the mean ages between the patients and control groups (*p* = 0.7630) (Table 1).

**Table 1:** Demographic characteristics of the study population

	*Control*	*Patients*	*p*
***Number***	40	55	
*Age*	36.2 ± 11.2	35.5 ± 10.8	0.763 (t test)
*Gender*	15 Males (37.5%) 25 Females (62.5%)	20 Males (36.4%) 35 Females (63.6%)	0.9097 (Chi square test)
*Type*		30 RR (54.6%) 20 SP (36.4%) 5 PP (9.1%)	

Note: Age is presented as mean ± SEM

MS Patients were assigned to one of three groups, depending on the clinical course of the disease. The groups were (i) relapsing-remitting (RRMS) (30; 55.6%), (ii) secondary progressive (SPMS) (20; 36.4%), and (iii) primary progressive (PPMS) (5, 9.1%). See Table 1. That is consistent with the literature, which indicates that about up to 85% of MS patient follow the RRMS course, and that more than half of those patients eventually develop SPMS, and that less than 10% of MS patients have a disease that is progressive from the onset (or PPMS) [33].

### HHV-6 antibody in MS patients

**Fig. 1:**
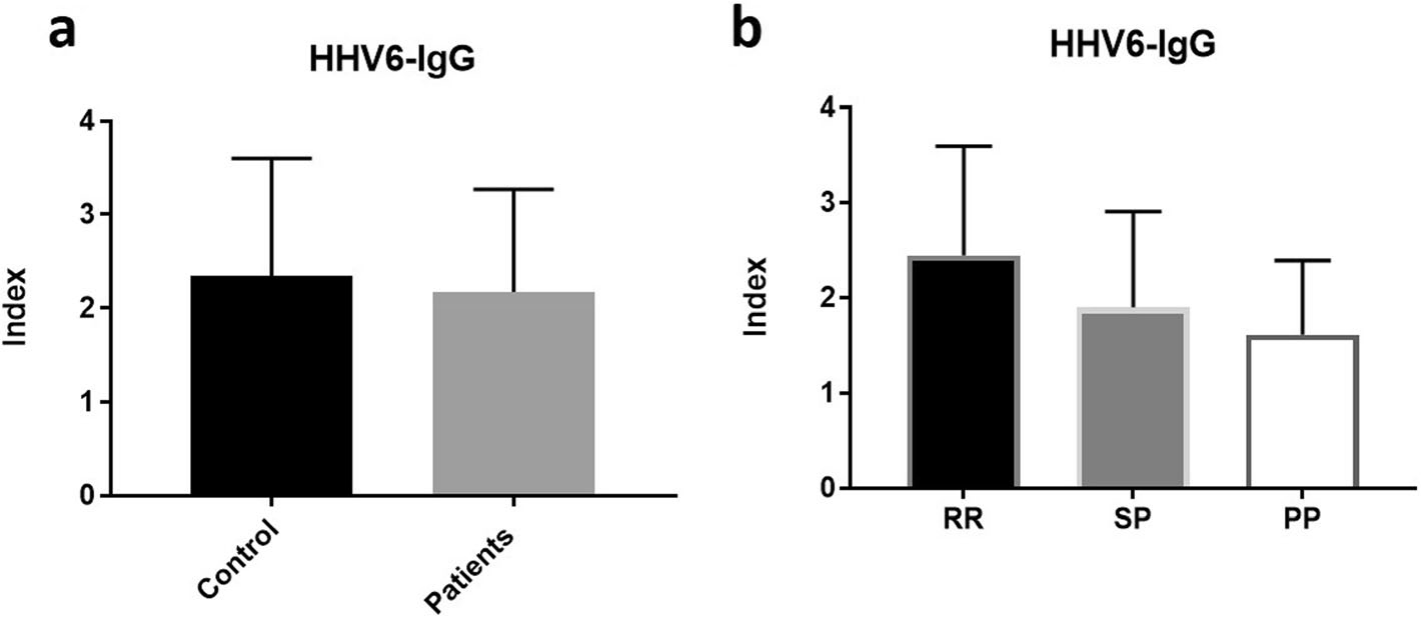
HHV-6 IgG levels: **a** shows a comparison in the levels of HHV-6 IgG between control and patients groups. **b** Shows a comparison in the HHV-6 IgG levels between the three types of MS (RRMS, SPMS and PPMS). The data is presented as the mean HHV6-IgG level ± SD

HHV-6 IgG antibody levels were measured for each all patients and controls, and an index value for each enrollee was calculated. Index values > 1.1 were considered positive (as per the instructions of the kit manufacturing company). There was no significant difference between patients and controls (86% of patients and 85% of controls were seropositive (*p* = 0.951). The mean index value for HHV-6 IgG antibody for the patients’ group was 2.171 ± 0.1485, which was slightly lower than that for the control group, which was 2.35 ± 0.198. However, the difference was not statistically significant (*p* = 0.4636) (Fig. 1a). Furthermore, there was no significant difference in HHV-6 IgG levels between the three types of MS (*p* = 0.1102). The mean index values were 2.446 ± 0.2098, 1.897 ± 0.2261 and 1.614 ± 0.3472 for RRMS, SPMS and PPMS, respectively (Fig. 1b).

### EBV antibodies in MS patients

Two EBV antibodies were measured; viral capsid antigen (VCA) IgG and EBV nuclear antigen-1 (EBNA) IgG in patients and controls using ELISA. Both antibodies, typically appear during or after the acute phase of infection and remain elevated for life. There was statistically insignificant difference in the prevalence of EBV antibodies between MS patients and controls (*p* = 0.755). As indicated in Table 2, The vast majority of patients (96%) and controls (98%) show evidence of previous exposure to EBV and tested positive for either VCA IgG, EBNA-1 IgG, or both.

**Table 2:** Prevalence of HHV-6, EBV and VZV IgG antibodies in the study populations. *p* < 0.05 is considered significant (Chi square test)

	Patients No. (%) of seropositive	Controls No. (%) of seropositive	***p***
**HHV-6 IgG**	**47 (86%)**	**34 (85%)**	**0.951**
**EBV IgG**			
**VCA IgG**	**50 (91%)**	**37 (93%)**	**0.783**
**EBNA-1 IgG**	**41 (75%)**	**31 (78%)**	**0.740**
**VCA IgG, EBNA-1 IgG or both**	**53 (96%)**	**39 (98%)**	**0.755**
**VZV IgG**	**54 (98%)**	**31 (78%)**	**0.001**

**Fig. 2:**
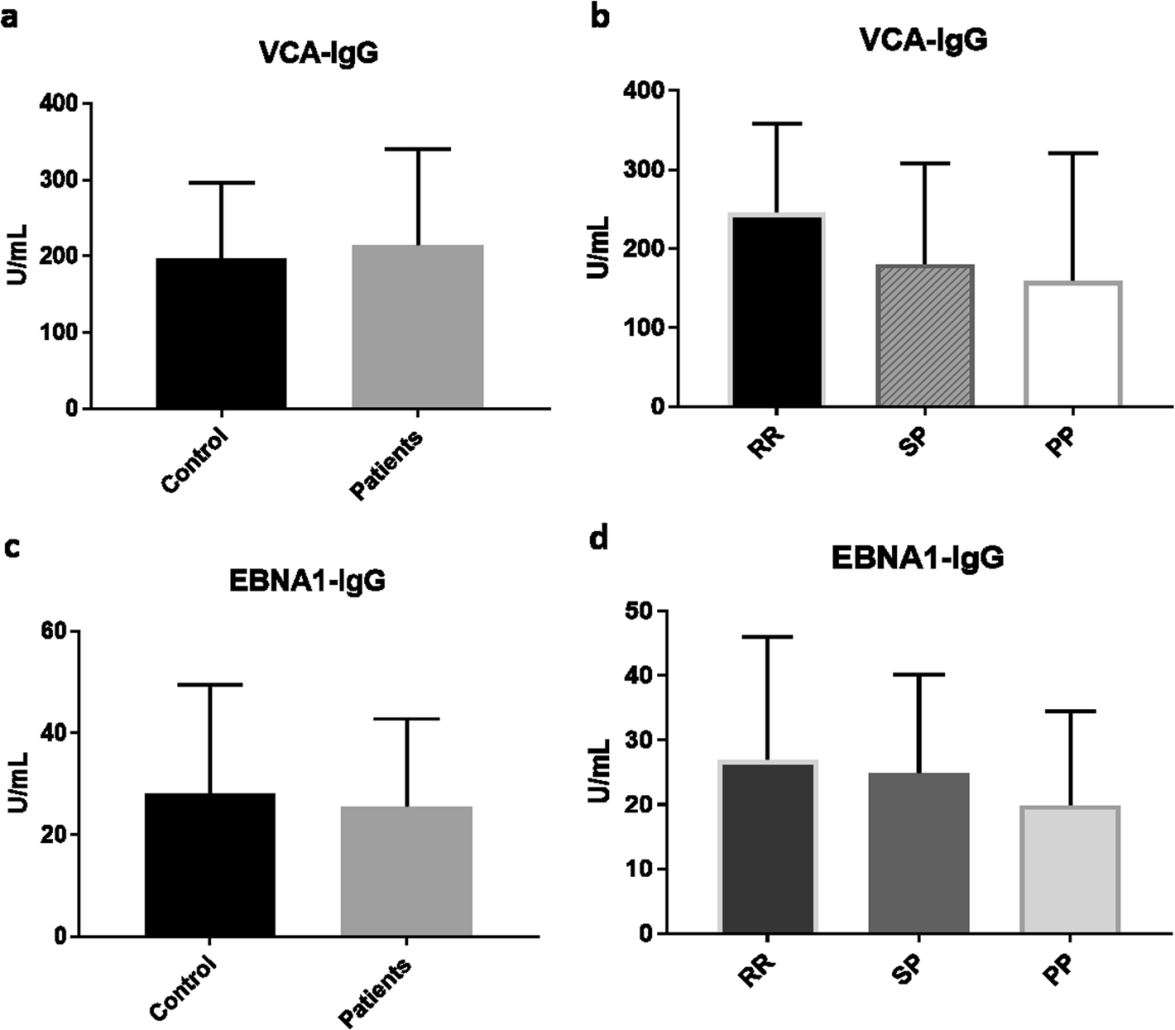
EBV antibody levels in MS patients: **a** VCA IgG antibody level in MS patients compared to control. **b** VCA IgG antibody levels in RRMS, SPMS and PPMS. **c** EBNA1 IgG antibody level in MS patients compared to control. **d** EBNA1 IgG antibody levels in RRMS, SPMS and PPMS. Data is presented as mean ± SD

As shown in Fig. 2, the mean for VCA levels were 214.2 ± 16.99 U/ml and 196.7 ± 15.77 U/ml for the patients and control groups, respectively. There was no statistically significant difference in VCA IgG level between the two groups (*p* = 0.47). The mean VCA levels in each type of MS were 245.9 ± 20.68 U/ml, 180.4 ± 28.66 U/ml and 159.1 ± 72.37 U/ml for the RRMS, SPMS and PPMS, respectively. Comparison of VCA IgG levels between the three types of MS did not show any statistically significant difference (*p* = 0.1160, one-way ANOVA test).

Similarly, there was no statistically significant difference in the EBNA1 IgG levels between patients’ and control group (*p* = 0.4861). The mean EBNA1 levels were 25.54 ± 2.322 U/ml and 28.3 ± 3.352 U/ml for the patients and control groups, respectively (Fig. 2 c). Finally, there was no statically significant difference in EBNA1 IgG levels between the three types of MS (*p* = 0.6878). The mean EBNA1 IgG levels for RRMS, SPMS and PPMS were 26.94 ± 3.48 U/ml, 24.85 ± 3.401 U/ml and 19.88 ± 6.527 U/ml respectively (Fig. 2).

### VZV antibody levels in MS patients

**Fig. 3:**
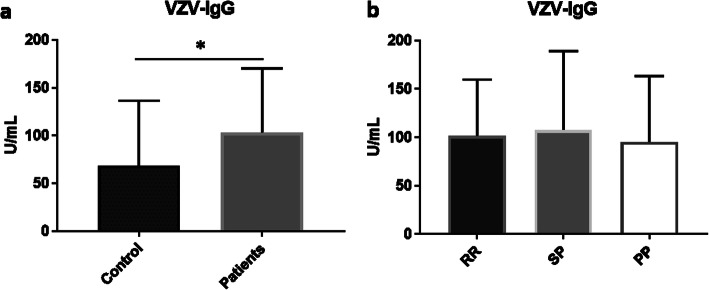
VZV antibody levels in MS patients: **a** VZV IgG antibody level in MS patients compared to control. **b** VZV IgG antibody levels in RRMS, SPMS and PPMS. Data is presented as mean ± SD

Finally, we also analyzed the prevalence and levels of VZV IgG antibody in MS patients. About 98% of patients tested positive for VZV IgG, which was significantly higher than the prevalence of VZV IgG (78%) in the controls (*p* = 0.001) (Table 2). By comparing the levels of VZV IgG between MS patients and controls, we found a significantly higher mean level of VZV IgG in MS patients (103.2 ± 9.052 U/ml) than the control (68.58 ± 10.73 U/ml) (*p* = 0.0152) (Fig. 3a). However, the levels of VZV IgG antibodies between the three types of MS did not show a statistically significant difference (*p* = 0.9177, one-way ANOVA test). The VZV IgG levels were 101.6 ± 10.6 U/ml, 107.7 ± 18.23 U/ml and 95.19 ± 30.35 U/ml for RRMS, SPMS and PPMS, respectively (Fig. 3b).

## Discussion

Understanding the etiology and pathogenesis of MS has been the focus of several research groups. Despite the extensive research, the exact etiology of MS remains ambiguous. However, nowadays, it is mainly acceptable that multiple genetic and environmental factors participate in the initiation and progression of the disease [34, 35]. Many considered exposure to viruses as one of the major etiologic environmental factors for MS [35]. This study is the first exploratory study to investigate the associated between viral exposure or reactivation and MS in the Jordanian population. The sample size was limited by the recent establishment of the local registry and availability of electronic medical records for MS patient in the north of Jordan. Future studies will be more likely to include larger number of patients as electronic medical records have become more readily available on a larger number of MS patients.

In this study, we investigated the exposure of Jordanian MS patients to three important viruses of the *Herpesviridae* family; HHV-6, EBV and VZV, by measuring serum levels of IgG antibodies to each of these viruses. Our results showed a higher prevalence and levels of VZV IgG antibody in MS patients compared to controls. This result is in agreement with previous reports from other geographical areas in which higher prevalence of VZV virus infection was also reported [36–40]. One published study was done in a middle eastern country by Aramideh Khouy et al. had also showed that the prevalence of VZV IgG antibodies was higher in MS patients than controls [41]. These results suggest a possible role of VZV infection in the etiology and/or pathogenesis of MS. The implication of VZV in MS is supported further by other studies in which VZV DNA was detected in the CSF and in the peripheral mononuclear cells of most MS patients during the relapse, but only in few of the patients during remission [42–45]. Furthermore, VZV-like viral particles were visualized by electron microscopy in the CSF of MS patients during relapse and these viral particles were infective to Vero E6 cells in vitro [45–47].

Primary infection with VZV causes varicella (chickenpox), after which the virus undergoes latency in the ganglia of peripheral somatic, autonomic, and enteric neurons [48]. Reactivation of the virus later in life causes a more serious disease known as zoster (shingles). The mechanism by which VZV infection participates in the development of MS is not clear [49]. Generally, several mechanisms of pathogen-induced autoimmunity were proposed including molecular mimicry, epitope spreading and bystander activation (reviewed in [49]). Molecular mimicry between viral and self-antigens may trigger a damaging autoimmune response against the myelin. In support of this hypothesis, glycoprotein E (gE) of VZV was reported to share more than 62% of the amino acid sequence with PrLD/M9 epitopes of the RNA-binding protein HNRNPA1 [50]. Due to this mimicry, antibodies and T-cell responses against viral gE my cross-react with HNRNPA1 proteins in the neurons leading to autoimmune responses against them.

The EBNA1 IgG levels were higher in the control groups than in the patients’ group. However, the difference was not statistically significant. In addition, the prevalence and HHV-6 antibody level between MS patients and controls was not statistically significant. These results must be interpreted with caution as they are in conflict with multiple reports from other countries [51–53]. The absence of association between MS and EBV seropositivity in our study could be attributed to the extremely high seropositivity rate in the normal control population (98%). This high prevalence is in agreement with other reports in the Middle East region [54, 55]. Such a high prevalence in normal control could mask any difference seen in MS patients. To overcome this, future studies should be conducted in Jordan with a much higher sample size.

## Conclusion

In summary, our study demonstrated statistically significant higher levels of VZV IgG in patients with MS compared to the control group in the north of Jordan which reflects the general population of Jordan, which is largely understudied. This finding may indicate that the previous infection with VZV may play a role in the etiology or the pathogenesis of MS in this population. Further studies are needed on the population of Jordan and the greater middle east in larger Cohorts to evaluate further the prevalence of the previous infection with VZV in patients with MS.

## Supplementary Information

**Additional file 1.**

## Data Availability

The datasets during and/or analysed during the current study available from the corresponding author on reasonable request.

## Abbreviations

MS: Multiple Sclerosis
EBV: Epstein-Barr Virus
HHV-6: Human herpesvirus 6
VZV: Varicella-Zoster Virus
CMV: Cytomegalovirus
CNS: Central Nervous System
RRMS: Relapsing-Remitting Multiple Sclerosis
SPMS: Secondary Progressive Multiple Sclerosis
PPMS: Primary Progressive Multiple Sclerosis
IgG: Immunoglobulin G
ELISA: Enzyme-linked Immunosorbent Assay
EBNA: Epstein-Barr nuclear antigen
VCA: Viral capsid antigen
SLE: Systemic lupus erythematosus
IRB: Institutional review board
