# Supplementary material for: Prevalence of seropositivity of selected herpesviruses in patients with multiple sclerosis in the North of Jordan
Source: BMC Neurol. 2020 Oct 29;20:397. doi: 10.1186/s12883-020-01977-w (PMC7596955; doi:10.1186/s12883-020-01977-w)
Supplement: Supplementary file 1 — Additional file 1. [file 12883_2020_1977_MOESM1_ESM.docx]

Sample Number: Date:

Please note that your answers will be confidential.

Age:

Gender: Male Female.

Date of diagnosis of Multiple Sclerosis.

What symptoms you suffered from and lead to the diagnosis of Multiple Sclerosis:

…………………………………………………………………………………………………………………………………………………….

Do you have any other chronic diseases? Yes No

If your answer is yes, please report them down:

……………………………………………………………………………………………………………………………………………………

Current treatment for Multiple Sclerosis: ……………………………………………………………………………………

Previous treatments for Multiple Sclerosis: ………………………………………………………………………………

Any other medications that you are currently taking: ……………………………………………………………….

……………………………………………………………………………………………………………………………………………………

Have any of your family members been diagnosed with Multiple Sclerosis? Yes No

If yes, what is the relation of that patient to you?...........................................................................

Has multiple Sclerosis caused any impairment in your daily activities? Yes No

Smoking status: Smoker None smoker.

Have you suffered any Multiple Sclerosis relapses in the last 2 years? Yes No

If your answer is yes, please explain: …………………………………………………………………………………………

…………………………………………………………………………………………………………………………………………………….

Your occupation: …………………………………………………………………………………………………………………………

The town or city where you live? ………………………………………………………………………………………………..

Thank you for your cooperation.
